# Left Atrial Enlargement on Transthoracic Echocardiography Predicts Left Atrial Thrombus on Transesophageal Echocardiography in Ischemic Stroke Patients

**DOI:** 10.1155/2016/7194676

**Published:** 2016-10-16

**Authors:** James Anaissie, Dominique Monlezun, A. Seelochan, James E. Siegler, Maria Chavez-Keatts, Jonathan Tiu, Denise Pineda, Alexander George, Amir Shaban, Nidal Abi Rafeh, Laurie Schluter, Sheryl Martin-Schild, Ramy El Khoury

**Affiliations:** ^1^Tulane Stroke Research Program, Tulane University Medical Center, New Orleans, LA, USA; ^2^Meharry Medical College, Nashville, TN, USA; ^3^Department of Neurology, Louisiana State University Health Sciences Center, New Orleans, LA, USA; ^4^Department of Neurology, University of Iowa, Iowa City, IA, USA; ^5^Department of Cardiology, Tulane University Medical Center, New Orleans, LA, USA

## Abstract

*Background.* Transesophageal echocardiogram (TEE) is superior to transthoracic echocardiogram (TTE) in detecting left atrial thrombus (LAT), a risk factor for stroke, but is costly and invasive, carrying a higher risk for complications.* Aims. *To determine the utility of using left atrial enlargement (LAE) on TTE to predict LAT on TEE.* Methods.* AIS patients who presented in 06/2008–7/2013 and underwent both TTE and TEE were identified from our prospective stroke registry. Analysis consisted of multivariate logistic regression with propensity score adjustment and receiver operating characteristic (ROC) area under the curve (AUC) analyses.* Results.* 219 AIS patients underwent both TTE and TEE. LAE on TTE was detected in 113 (51.6%) of AIS patients. Patients with LAE on TTE had higher proportion of LAT on TEE (8.4% versus 1.0%, *p* = 0.018). LAE on TTE predicted increased odds of LAT on TEE (OR = 8.83, 95% CI 1.04–74.83, *p* = 0.046). The sensitivity and specificity for LAT on TEE by LAE on TEE were 88.89% and 52.20%, respectively (AUC = 0.7054, 95% CI 0.5906–0.8202).* Conclusions.* LAE on TTE can predict LAT detected on TEE in nearly 90% of patients. This demonstrates the utility of LAE on TTE as a potential screening tool for LAT, potentially limiting unneeded costs and complications associated with TEE.

## 1. Introduction

Approximately one in four acute ischemic strokes (AIS) can be attributed to an embolism that originates in the heart [1, 2]. In particular, cardiac morphology and rhythm changes have been documented as chief contributing factors for thrombus formation [3–6]. The left atrium is the most common chamber in which these thrombi originate, and thrombus formation is strongly associated with the pathophysiology of atrial fibrillation [4, 5]. The disease process leads to rhythmic and structural changes within the heart, with left atrium dilation and left atrium appendage (LAA) dysfunction being prominent contributing factors to thrombus formation in these patients [5].

As left atrial enlargement (LAE) is thought to be a structural precursor of atrial fibrillation, accurate detection of this cardiac abnormality can aid as a screening modality for prevention of recurrent AIS. To properly assess such cardiac changes, echocardiography serves an important diagnostic tool for at-risk patients. However, the decision to perform transesophageal echocardiography (TEE) after routine transthoracic echocardiography (TTE) in AIS is controversial.  Table 1 lists some of the common indications for choosing one of the two echocardiographic imaging modalities.

Prior literature and clinical practice have recognized the cost-effectiveness and risk TEE or TTE in patients with suspected cardiac disease [3, 4]. TTE is noninvasive and less expensive than TEE; conversely, TEE is superior to TTE in detecting cardiac structural abnormalities and intracardiac thrombi [3, 4]. Specifically, TEE is thought to be superior at quantifying the degree of LAE and visualizing the LAA. As both the LAE and LAA are considered to be important sites and/or precursors of thrombi formation in cardioembolic stroke, TEE is considered to have a higher utility for patients who are at high risk for AIS (such as prior history of embolic stroke, AF, and valvular abnormalities). Further complicating the decision making between these modalities is the fact that TEE is not tolerated by all patients and carries a higher risk of inpatient complications—including bleeding and transient cardiac arrhythmias—which may lead to extended hospital stay [6].

Due to the potential aforementioned disadvantages found in TEE, it is worthwhile to compare the diagnostic and/or screening value of TTE versus TEE. In this study, we sought to determine the utility of TTE imaging in AIS patients. Our first analysis centers around the use of TTE to diagnose LAE in these patients as opposed to the standard TEE imaging. Secondly, the ability for TTE to detect left atrial thrombus (LAT) within AIS patients that have evidence of LAE will be evaluated by comparing against patients who had a LAT found on TEE (the gold standard for detecting LAT).

## 2. Methods

### 2.1. Study Population

We conducted a single-center retrospective analysis of all consecutive patients who presented with AIS between July 2008 and July 2013 [7]. Patients who did not have both TTE and TEE were excluded. Stroke etiology was defined according to the Trial of Org 10172 in Acute Stroke Treatment [8]. Patient demographics, past medical history, and imaging characteristics were all collected prospectively in our stroke registry. All patients were assessed by staff stroke neurologists or stroke fellows at a single academic emergency department.

### 2.2. Definitions

Patients that were included within the retrospective analysis were classified as having LAE on the basis of left atrial volume to body surface area measurements noted from TTE and TEE imaging. As per recommendations from the American Society of Echocardiography, the ratio of left atrial volume to body surface area was used for the evaluation on TTE. For TEE, evaluation and classification were done on the basis of the biplane area formula noted in Lang et al. [9]. The patients were divided into 2 groups: (1) no LAE (LA volume to BSA ratio <34 mL/m^2^ on TTE) and (2) LAE (moderate LAE noted to have measured ratios of 34–39 mL/m^2^ on TTE; severe LAE defined as LA volume to BSA ratio >39 mL/m^2^ as per Tsang et al.) [10].

### 2.3. Statistical Analyses

The patient demographics, past medical history, and imaging characteristics were compared between two groups (Table 2). Namely, AIS patients with evidence of LAE on the screening TTE (*n* = 113) were compared against their counterparts without LAE identified via TTE (*n* = 106). To maintain standardization between the two groups, the individual's chief clinician was chiefly responsible for guiding the decision to obtain both diagnostic tests. Subsequently, both of the analyzed groups were narrowed specifically to patients who underwent both TTE and TEE. The comparative metrics were in line with criteria used for choosing either TTE or TEE as an echocardiographic modality [11, 12].

Categorical data, presented as proportions, were analyzed using Pearson Chi-Square or Fisher exact test where appropriate. Continuous data, presented as medians and ranges, were compared using Wilcoxon Rank Sum tests. Univariate logistic regression was used to assess the odds of predicting LAT on TEE for each group. Receiver operator characteristic (ROC) curves were generated to assess the ability of LAE on TTE to predict LAT on TEE. No adjustments were made for multiple comparisons as this was an exploratory analysis.

Propensity score (PS) matching was conducted based on the likelihood of receiving TEE among TTE subjects to control for the confounding effect of baseline patient traits associated with receiving TEE and TTE. The PS was created based on documented predictors of receiving TEE, regardless of their statistical significance on bivariate analysis, to create a parsimonious probit regression model predicting treatment [13, 14].

Multivariate logistic regression with adjustment for the PS was then performed for LAE on TTE predicting LAT on TEE. Adjustment is a popular PS technique in stroke outcome research that has been shown to be similar to other PS techniques of matching and weighting [15]. Outcome results were then compared to average treatment effect on the treated (ATT) between PS-matched subjects using Kernel matching with imposition of common support [16, 17].

## 3. Results

Among the 219 AIS patients who had both TTE and TEE performed, LAE on TTE was detected in 113 patients (51.6%). Patients with LAE on TTE were more likely to be older (64 years versus 59 years, *p* < 0.001) and were more likely to have a history of chronic heart failure (18.81% versus 1.94%, *p* < 0.001) or hypertension (86.36% versus 68.57%, *p* = 0.002) when compared to patients with normal left atrial size.

Table 3 displays the differences in TEE findings for the two study groups. Patients with LAE on TTE exhibited higher median left atrial volume to BSA ratio compared to patients without LAE on TTE (4.2 mL/m^2^ versus 3.4 mL/m^2^, *p* < 0.001). Patients with LAE on TTE were more likely to have evidence of valvular disorders, which included mitral valve regurgitation (54.86% versus 30.18%, *p* < 0.001), mitral annular calcification (46.81% versus 21.43%, *p* < 0.001), or aortic valve stenosis (31.25% versus 16.04%, *p* = 0.004). Patients with LAE on TTE were also more likely to have left ventricular dilatation (17.02% versus 4.04%, *p* = 0.003) and left ventricular hypertrophy (54.93% versus 19.12%, *p* < 0.001) on TEE than patients without LAE.

Among the entire sample, TTE failed to detect LAT whereas 9 patients (4.11%) had LAT on TEE. Among patients with both TTE and TEE documenting presence or absence of LAE, LAE was detected in 89 patients by TTE and 76 by TEE, with LAE detected by both TTE and TEE in 63 (70.79%) patients, TTE alone in 26 (29.21%), and TEE alone in 13 (15.29%), as seen in Figure 2. As a result, TTE was 89.68% sensitive for detecting LAE whereas TEE was 57.94% sensitive. Moreover, the patients who showed evidence of LAE on TTE were more likely to show signs of thrombus on TEE imaging (8.4% versus 1.0%, *p* = 0.018).

ATT results of PS-matched subjects showed a similarly increased likelihood of LAT when LAE was detected using TTE (7.5%, *T*-stat = 2.12, *p* < 0.05). Within the subgroup of patients who displayed evidence of LAE on TTE (*n* = 113), 8 (8.42%) had LAT on TEE. Therefore, TEE conferred a PPV value of 92.9% (8/9) in detecting LAT. Conversely, the absence of a LAE on TTE correlated with NPV of 99% for detecting LAT (105/106).

**Figure 1:**
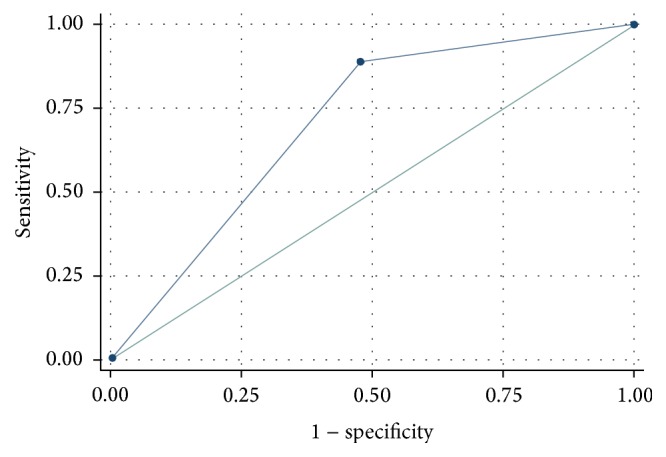
Receiver operating characteristic curve of left atrial enlargement on transthoracic echocardiogram classifying left atrial thrombus on transesophageal echocardiogram. Area under curve = 0.7054 (95% CI 0.5906–0.8202).

**Figure 2:**
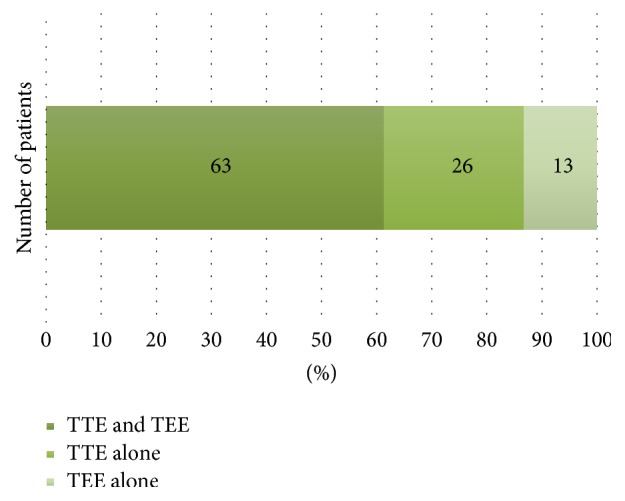
Number of patients demonstrating left atrial enlargement on each echocardiographic imaging modality. TTE = transthoracic echocardiogram; TEE = transesophageal echocardiogram.

The ROC curve for LAE on TTE predicting LAT is displayed in Figure 1. The finding of LAE on TTE predicted LAT with a sensitivity of 88.9% and specificity of 52.2% (ROC = 0.7054, 95% CI 0.5906–0.8202). Additionally, multivariate logistic regression with adjustment for the PS revealed that LAE on TTE was associated with an increased odds of LAT detection (OR = 8.83, 95% CI 1.04–74.83, *p* = 0.046).

## 4. Discussion

The results of our study indicate that a normal left atrium on TTE practically excludes the possibility of thrombus formation in this cardiac chamber. As the finding of LAE on TTE was almost 90% sensitive for predicting LAT on TEE, we believe that performing a TTE is a sufficient screening modality for detecting LAT in patients with AIS. While our results support prior investigations which confirmed the utility of TEE in detecting LA [18], the risk of conducting a transesophageal study is not inconsequential. Although life-threatening sequelae are rare (<2%), intraoperative risks are notable for cardiac arrhythmias, hypoxia, hypotension, adverse response to anesthesia, and esophageal or gastric perforation while postoperative complications include odynophagia, dysphagia, and transient or permanent vocal cord dysfunction [6, 19–21]. Our findings support that LAT is unlikely to be found on TEE if TTE shows normal LA size.

Our study is unique in that we analyzed the utility of TTE and TEE for AIS patients through a robust propensity score matching technique. This can reduce the selection bias added from choice of imaging modality, as well as the influence of possible confounding factors from an observational study design. This methodology, which was used for a similar reason by a recent multicenter study [22], can better approximate the more unbiased results seen in a randomized controlled trial. A randomized trial otherwise may not be performed due to the inherent challenges of implementing a randomized approach to diagnostic imaging for patient care. As a result, our study can be viewed as a meaningful contribution to ameliorate the gap in the literature on this topic.

Although our findings are not unexpected, the implications are considerable. By utilizing TTE to screen for LAT, providers have the opportunity to cut the complications and costs associated with routine TEE. For stroke patients in whom a left atrial thrombus is suspected, a normally sized left atrium on transthoracic echocardiogram may be sufficient if windows are adequate and the technician is experienced. Advances in technology and technique have improved the yield of this diagnostic modality [23], and newer devices may permit more mobile and accurate data capture [24]. Knowledge that TTE may be sufficient for predicting LAT formation may improve the confidence of providers who are considering TEE for patients at greater risk of perioperative complications.

In our study, we found that only about 71% of patients who had LAE on TTE had confirmatory LAE on TEE. Biplane axial length measurement is the recommended method of determining left atrial size on TEE; however, it often gives the largest value for the calculation for left atrial volume [25]. Although TEE is considered to be the gold standard for visualization of left atrial abnormalities [12], the appearance of thrombi formation among patients can vary [6, 26]. Thus, intraoperator imaging and interpretation may contribute to the analyses conducted within our study. Moreover, due to the population served by the Stroke Center, a wide age range was noted in both groups involved in this study. As a result, the younger patient population in both groups may possibly preclude the efficacy of TTE to analyze cardiac dimensions or contents properly [27, 28], especially given the superior aspect of the cardiac chamber analyzed within this study.

While LAE and its various relationships to AIS have been reviewed throughout this report, the relationship between cardiac dimensions noted on echocardiographic imaging and clinically relevant endpoints has been recently established within the literature. For patients with AF, actual chamber size (measured in cm^2^ on TEE) has not been associated with the development of embolic events or AIS [29]. However, the volume to BSA ratio (cm/m^2^), thrombi size, and LAA size have been noted to be important factors in the formation of thrombi in AIS patients [27–29].

Our study is limited by its small sample size representing patients from a single center. As a result, the low LAT event rate may have prevented sufficient comparison between TTE and TEE parameters in predicting this outcome. There is also potential for selection bias as to which patients had both TTE and TEE performed. At our institution, the decision to perform TTE and/or TEE is individually made for each patient by the treating physician, and some patients with equivocal findings on TTE may have had TEE ordered secondarily to pursue cardioembolic causes of stroke. However, considering the limitations of a cross-sectional design, propensity score matching is a rigorous and sophisticated way of reducing selection bias as much as possible by controlling for probability of receiving TTE versus both TTE and TEE. We did not explore other TEE indications for oral anticoagulant therapy such as “smoke”/spontaneous echo contrast. For this reason, one cannot conclude that TEE has no value in the evaluation of the cause of stroke in patients without LAE on TTE. We can only conclude that LAT is unlikely to be found on TEE if TTE shows normal LA size.

In conclusion, our results may help increase provider confidence for using TTE in the AIS patient. Since TEE is highly sensitive, initial testing can be done with this modality if there are acute indications of AIS in a patient. With TTE evidence of LAE as a prerequisite for TEE, providers may detect the presence of LAT in patients with AIS in a safe and effective manner.

## Figures and Tables

**Table 1:** Indications for transthoracic echocardiography versus transesophageal imaging for embolism detection (adapted from [12]).

TTE	TEE
Patients ≥45 years with a neurologic event and no identified cerebrovascular disease	Patients <45 years without known cardiovascular disease (i.e., absence of infarction or valvular disease history)
Any patient with an abrupt occlusion of a major peripheral or visceral artery	Patients with a high pretest probability of a cardiac embolic source in whom a negative TTE would be likely to be falsely negative
Patients with a high suspicion of left ventricular apical thrombus	Patients with atrial fibrillation and suspected left atrial or left atrial appendage thrombus, especially in the absence of therapeutic anticoagulation
Patients in whom TEE is contraindicated (e.g., esophageal stricture, unstable hemodynamic status) or who refuse TEE	Patients with a mechanical heart valve
	Patients with suspected aortic pathology

**Table 2:** Clinical and demographic characteristics of AIS patients with and without left atrial enlargement on transthoracic echocardiogram.

	AIS subjects without LAE on TTE *n* = 106 (48.4%)	AIS subjects with LAE on TTE *n* = 113 (51.6%)	*p* value
Age, median (range)	59 (26–83)	64 (26–90)	<0.001
African American, number (%)	74 (71.15%)	73 (66.36%)	0.450
Past Medical History, number (%)			
Atrial fibrillation	4 (3.81%)	11 (10.00%)	0.075
Chronic heart failure	2 (1.94%)	19 (18.81%)	<0.001
Diabetes	25 (24.51%)	40 (37.04%)	0.050
Hypertension	72 (68.57%)	95 (86.36%)	0.002
Coronary artery disease	16 (15.09%)	27 (24.77%)	0.076
Known prior stroke	35 (33.33%)	39 (35.78%)	0.707
Hyperlipidemia	31 (29.52%)	42 (38.18%)	0.180
Carotid stenosis	0 (0.00%)	0 (0.00%)	NA
Active smoker	43 (41.75%)	40 (36.70%)	0.451

Definitions: LAE, left atrial enlargement; TTE, transthoracic echocardiogram.

**Table 3:** Transesophageal echocardiographic determination of cardiac characteristics of AIS patients (results stated with number of patients observed with noted characteristic on TEE; percentage noted subsequently is a comparison to the number of patients who have a certain characteristic on TTE).

	AIS subjects without LAE on TTE *n* = 106 (48.4%)	AIS subjects with LAE on TTE *n* = 113 (51.6%)	*p* value
Left atrial size, median (range), mL/m^2^	3.4 (2.3–13.0)	4.2 (2.7–6.7)	<0.001
Left ventricular size, median (range), mL/m^2^	89 (44–303)	98 (31.6–1333)	0.081
TTE detection of LAT, %	0 (0.00%)	0 (0.00%)	NA
PFO, %	1 (1.32%)	3 (3.85%)	0.324
ASD, %	29 (27.89%)	30 (28.30%)	0.052
Interatrial shunt, %	5 (5.95%)	10 (11.24%)	0.217
Mitral valve regurgitation, %	32 (30.18%)	62 (54.86%)	<0.001
Mitral valve prolapse, %	0 (0.00%)	2 (3.12%)	0.136
Mitral annular calcification, %	18 (21.43%)	44 (46.81%)	<0.001
Left ventricular dilatation, %	4 (4.04%)	16 (17.02%)	0.003
Left ventricular hypertrophy, %	13 (19.12%)	19 (54.93%)	<0.001
Diastolic dysfunction, %	59 (62.11%)	71 (83.53%)	0.001
Aortic valve regurgitation, %	28 (26.42%)	42 (38.19%)	0.230
Aortic valve stenosis, %	17 (16.04%)	35 (31.25%)	0.004
TEE LAE, %	13 (15.29%)	63 (70.79%)	<0.001
TEE detection of LAT, %	1 (1.04%)	8 (8.42%)	0.016

Key: LAE, left atrial enlargement; PFO, patent foramen ovale; ASD, atrial septal defect; TTE, transthoracic echocardiogram; TEE, transesophageal echocardiogram; LAT, left atrial thrombus; NA, not applicable.
